# PG-SGA SF in nutrition assessment and survival prediction for elderly patients with cancer

**DOI:** 10.1186/s12877-021-02662-4

**Published:** 2021-12-10

**Authors:** Qi Zhang, Xiang-Rui Li, Xi Zhang, Jia-Shan Ding, Tong Liu, Liang Qian, Meng-Meng Song, Chun-Hua Song, Rocco Barazzoni, Meng Tang, Kun-Hua Wang, Hong-Xia Xu, Han-Ping Shi, Investigation on Nutrition Status and its Clinical Outcome of Common Cancers (INSCOC) Group

**Affiliations:** 1 Department of Gastrointestinal Surgery, Beijing Shijitan Hospital, Capital Medical University, Beijing, 100038 China; 2 Department of Clinical Nutrition, Beijing Shijitan Hospital, Capital Medical University, Beijing, 100038 China; 3 Beijing International Science and Technology Cooperation Base for Cancer Metabolism and Nutrition, Beijing, 100038 China; 4 Capital Medical University, Beijing, 100038 China; 5 Department of Obstetrics and Gynecology, the First Affiliated Hospital of Nanchang University, Nanchang, 330006 Jiangxi China; 6 Department of Obstetrics and Gynecology, Hangzhou Women’s hospital/ Hangzhou Maternal and Child Health Hospital/ Hangzhou First People’s Hospital Qianjiang New City Campus, Hangzhou, 310008 China; 7 Department of Epidemiology, College of Public Health, Zhengzhou University, Zhengzhou, 450001 Henan China; 8 Department of Medical, Surgical and Health Sciences – University of Trieste, Trieste, Italy; 9 Department of Gastrointestinal Surgery, Institute of Gastroenterology, the First Affiliated Hospital of Kunming Medical University, Kunming, 650032 Yunnan China; 10 Department of Clinical Nutrition, Daping Hospital, Army Medical University, Chongqing, 400042 China

**Keywords:** Malnutrition, Cancer, Elderly patients, Nutrition assessment, PG-SGA SF

## Abstract

**Background:**

This study was sought to report the prevalence of malnutrition in elderly patients with cancer. Validate the predictive value of the nutritional assessment tool (Patient-Generated Subjective Global Assessment Short Form, PG-SGA SF) for clinical outcomes and assist the therapeutic decision.

**Methods:**

This is a secondary analysis of a multicentric, observational cohort study. Elderly patients with cancer older than 65 years were enrolled after the first admission. Nutritional status was identified using the PG-SGA SF.

**Results:**

Of the 2724 elderly patients included in the analysis, 65.27% of patients were male (*n* = 1778); the mean age was 71.00 ± 5.36 years. 31.5% of patients were considered malnourished according to PG-SGA SF. In multivariate analysis, malnutrition(PG-SGA SF > 5) was significantly associated with worse OS (HR: 1.47,95%CI:1.29–1.68), affects the quality of life, and was related to more frequent nutrition impact symptoms. During a median follow-up of 4.5 years, 1176 death occurred. The mortality risk was 41.10% for malnutrition during the first 12 months and led to a rate of 323.98 events per-1000-patient-years. All nutritional assessment tools were correlated with each other (PG-SGA SF vs. PG-SGA: r = 0.98; PG-SGA SF vs. GLIM[Global Leadership Initiative on Malnutrition]: r = 0.48, all *P* < 0.05). PG-SGA SF and PG-SGA performed similarly to predict mortality but better than GLIM. PG-SGA SF improves the predictive ability of the TNM classification system for mortality in elderly patients with cancer, including distinguishing patients’ prognoses and directing immunotherapy.

**Conclusions:**

The nutritional status as measured by PG-SGA SF which is a prognostic factor for OS in elderly cancer patients and could improve the prognostic model of TNM.

**Supplementary Information:**

The online version contains supplementary material available at 10.1186/s12877-021-02662-4.

## Background

Solid tumors remain the leading cause of cancer-related deaths worldwide. Improving the overall survival (OS) of patients is the most crucial target of anti-cancer therapy; thus, variables that predict the prognosis are clinical and investigative interest [1]. The TNM staging system described in the 8th American Joint Committee on Cancer (AJCC) Staging Manual is the most widely used one [2]. The current TNM staging system is essential for predicting clinical outcomes and determining appropriate treatments. However, the survival of patients varies among patients with the same disease stage, ranging from only a few weeks to several years [3]. Identifying high-risk patients with cancer based on changeable clinical characteristics is crucial to reducing the risk of mortality.

Patients with cancer are known to have a higher risk of malnutrition than those without cancer, especially elderly patients with cancer. Understanding nutritional status in elderly patients with cancer is essential to therapeutic decisions and their survival [4]. Compared to other clinical covariates, malnutrition has the advantage that it is a modifiable risk factor. According to nutrition assessments from several countries, 25 to 85% of cancer patients estimate to have cancer-related malnutrition [5]. More than half of all patients with solid tumors suffer from malnutrition, which is associated with decreased therapeutic response and increased mortality [6]. Nutritional status is closely associated with the survival and treatment of cancer patients [7]. In the past few years, one of the critical measures for improving the comprehensive clinical treatment for elderly patients with cancer is the nutritional assessment [8]. Older age is a well-known predictor of worse cancer survival [9]. There almost 70% of cancer death occurs in elderly patients [10]. Previous studies demonstrated a significant association between nutritional state and risk of death in elderly patients with cancer in the geriatric oncology setting. And, this affects only during the first few years after diagnosis [11]. In the study by Boulahssass et al., the nutritional status has greater weight in the patients with cancer, even more than tumor stage [10]. Therefore, when estimating the clinical outcome in cancer patients, various nutrition-related factors, in addition to the current TNM staging system, should be considered.

A variety of nutrition assessment tools, such as the Patient-Generated Subjective Global Assessment (PG-SGA), the Malnutrition Universal Screening Tool (MUST), the Global Leadership Initiative on Malnutrition (GLIM), andthe Mini Nutritional Assessment (MNA), are chosen in hospitals [12–16]. However, we have known that many nutritional assessment tools could not be entirely usable and applicable in actual clinical practice. The main reason is the relative scarcity of treatment physician resources and a lack of understanding of the nutritional assessment tools by both patients and physicians.

The Patient-Generated Subjective Global Assessment (PG-SGA) is a nutrition assessment tool based on the SGA.It is widely recommended by the Academy of Nutrition and Dietetics (AND) for cancer patients [12]. However, a standardized PG-SGA protocol could take too much time even if the interviewers were well trained. Some items on the PG-SGA may be perceived as complicated to comprehend by the patients or as challenging to perform by healthcare professionals, especially the physical exam [17]. Recently, the GLIM criteria were proposed as the malnutrition diagnosis standard in the clinical setting and call for validation. Our previous study validated the GLIM criteria for identifying malnutrition in the elderly oncology population and its predictive value regarding survival in the patients with oncology [18, 19].

Interestingly, the Patient-Generated Subjective Global Assessment Short Form (PG-SGA SF) received increasing attention as a professional nutritional assessment tool [20]. The PG-SGA SF is a component of the full PG-SGA, retains the patient-reported component (including weight, food intake, symptoms, activity, and function). Since it is designed to be patient-led, it is relatively simple to complete, and it could conserve time for both patients and physicians [20]. The predictive power of this assessment tool has qualified in various patients with cancer, including patients with incurable cancer, chemotherapy outpatients, and patients with head and neck cancer [21].

The current study aimed to validate the prognostic power of the PG-SGA SF in elderly patients with cancer. We also sought to report the prevalence and clinical associations of malnutrition in a contemporary cohort of elderly patients with cancer using PG-SGA SF, PG-SGA standards, and GLIM.

## Methods

### Study population and design

This study is a retrospective study based on the Investigation on Nutrition Status and its Clinical Outcome of Common Cancers (INSCOC) cohort in China; a detailed description of the design, methods and development of the INSCOC study was provided elsewhere [22, 23]. The patients with pathologically diagnosed solid tumor(s) at any stage who met the inclusion criteria were recruited from multiple institutions in China between 2013 and 2020. The inclusion criteria in the present study were: 1) patients aged 65 years or more; 2) a histological diagnosis of the solid malignant tumor; and 3) a hospital stay longer than 48 h. The exclusion criteria were: 1) patients with Acquired immunodeficiency syndrome (AIDS) or transplanted organ(s); 2) patients who were admitted to the intensive care unit (ICU) and were in a critical condition at the beginning of recruitment, 3) patients who refused to participate or would not cooperate with the questionnaire survey. Additionally, as shown in the study schematic (Supplementary Fig. 1), participants who had a missing critical clinical examination, or follow-up data, or more than 10% of all data, were excluded. Finally, 2724 elderly patients were included in the current analysis. The study was conducted in line with the Helsinki declaration; its design was approved by the local Ethics Committees of all participants’ hospitals. All patients signed an informed consent form before participating in the study. The trial was registered at http://www.chictr.org.cn with registration number ChiCTR1800020329.

### Malnutrition assessment

Body mass index (BMI) was calculated for all participants, categorized using the classifications for the Chinese population: underweight (< 18.5 kg/m2), normal weight (18.5 ~ 23.9 kg/m2), overweight, or obesity (> 24 kg/m2). Malnutrition was assessing used three nutritional assessment tools for all patients. First, participants were evaluated by dietitians using standard PG-SGA to determine their degree of malnutrition. The participants were classified into two categories: non-malnutrition (PG-SGA < 4); malnutrition (PG-SGA ≥ 4). Based on GLIM criteria, at least one phenotypic (weight loss (%) within 6 months, low BMI, and reduced muscle mass) and one etiologic (reduced food intake or assimilation, disease burden, and inflammatory condition of cancer) criterion were required to diagnose malnutrition when participants were screened at risk of malnutrition in NRS 2002 [19]. As all participants with cancer met the etiologic criterion of the GLIM criteria, it was excluded from the GLIM used in this study. The PG-SGA SF consists of four boxes: 1) body weight, 2) food intake, 3) symptoms affecting oral food intake, and 4) activities and function. According to the PG-SGA SF, the optimal cut-off value to determine malnutrition was five by using maximally selected rank statistics (supplementary Fig. 2).

### Data collection

The demographic, anthropometric, and clinical parameters were collected for all participants with the first 48 h after admission, including gender, age, BMI, primary tumor site, TNM stage, Chronic Disease information, lifestyle habits (e.g., alcohol, smoking), Karnofsky Performance Status (KPS). Pathological staging was defined according to the 8th edition of the AJCC TNM staging system. Treatment information and follow-up data were also collected. Fasting blood tests, such as albumin, globulin, creatinine, neutrophil, and lymphocyte, were collected with standard laboratory techniques within 48 h of admission. Albumin-globulin ratio (A/G) and neutrophil-lymphocyte ratio (NLR) were calculated, the NLR ≥ 3 was defined as elevated NLR in this study. The calf-circumference (CC) was measured using flexible and non-elastic tape. Handgrip strength (HGS) was measured in the dominant hand with a Jamar dynamometer.

### Patient outcome

The primary outcome was overall survival. Through the electronic medical record system, patients with one or more re-admission records could be extracted. The overall survival (OS) time was defined as participants who were followed up from the date of the first-time admission until death from any cause, or the end of follow-up (December 31, 2020), whichever came first. For overall mortality and the event rate per 1000 patient-years of follow-up, individuals alive at the end of follow-up were censored at that time.

### Statistical analysis

Variables are expressed as the means±standard (SD), percentage, or median with interquartile range. Their differences were analyzed using Student’s t-test to see if variables followed a normal distribution or nonparametric tests (Mann-Whitney or Kruskal-Wallis) if variables did not follow a normal distribution. Qualitative variables were analyzed using chi-square tests or Fisher corrections if necessary. Kaplan-Meier curves were used to analyze the survival data, and the Log-rank tests were used to compare survival between groups. Cox regression analysis was used to evaluate the prognostic impact of malnutrition, including those covariates associated with known poor prognosis or *p*-value < 0.05 in the univariate cox analysis. Adjusted hazard ratios (HRs) and their corresponding 95% confidence intervals (CIs) were derived from Cox models after adjusting for covariates.

The possible linear relationship between PG-SGA SF and the all-cause mortality was evaluated using restricted cubic spline regression. The time-dependent receiver operating characteristic (ROC) curves, area under the curve (AUC) analyses,and ROC-AUC values for each time-point were used to evaluate the predictive performance of the malnutrition assessment tools. The Harrell C-statistics, continuous net reclassification improvement (cNRI), and integrated discrimination improvement (IDI) were calculated to assess and compare the discrimination capacity of the PG-SGA SF to predict mortality. Calibration curves were generated by comparing the predicted survival with the observed survival after bias correction. To evaluate the potential clinical net benefit of the model, the researchers performed a decision curve analysis (DCA). The significance level was set at *P* < 0.05 (two-sided probability). All analysis was performed using R version 3.6.2 (http://www.rproject.org). DCA was performed using the source file “stdca.r”, downloaded from https://www.mskcc.org.

## Results

### Clinical features and characteristics of the study population

A total of 2724 elderly patients were included in the final analysis according to the screening criteria. Most patients were male (*n* = 1778; 65.27%), and the mean age was 71.00 ± 5.36 years. Digestive system cancers were the most common diagnosis (*n* = 1245; 45.70%). A large proportion of patients (*n* = 1117) had metastatic cancer. The majority of patients received or prepared at least one active treatment, including surgery (64.10%), chemotherapy (53.40%), radiotherapy (15.30%), or immunotherapy (4.88%). The percentage of patients with malnutrition varied from 31.5% with the PG-SGA SF, to 64.6% with the PG-SGA, and to 27.60% with the GLIM. More data on the baseline demographic and clinical characteristics of the study population are shown in Supplementary Table 1. Most of the patient characteristics and clinical parameters were statistically significant in univariate analysis. Both PG-SGA (1.31, 95%CI: 1.13–1.51), PG-SGA SF (1.47, 95%CI: 1.29–1.68), and GLIM (1.24, 95%CI: 1.07–1.44) were identified as significant predictors of overall survival (Supplementary Tables 2 and 3).

### Patient characteristics and clinical Association of Malnutrition

**Fig. 1:**
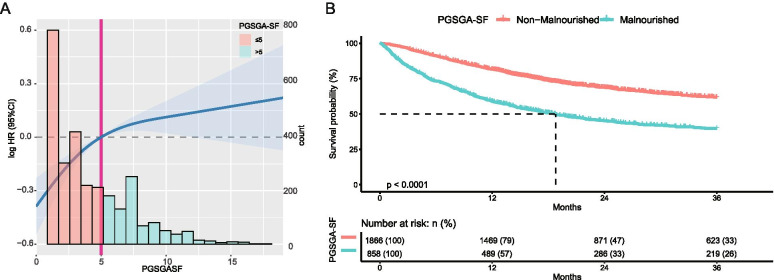
The relationship between PG-SGA SF and OS. **A** The incidence of all-cause mortality is shown after adjusted for gender, age, smoking, alcohol, tumors type, TNM stage, surgery, radiotherapy, and chemotherapy, KPS, A/B, NLR, and HGS. The x-axis shows the score of malnutrition indexes (PG-SGA SF). The curve shows the incidence, with 95% confidence intervals, of the estimates. Histograms show the population distribution of malnutrition indexes. **B** Kaplan-Meier curves for all-cause mortality by the cut-off point of PG-SGA SF (> 5) in elderly patients with cancer

Over a median of 4.5 years of follow-up, we observed 1176 deaths. The overall mortality rate for elderly patients with cancer at 12 months was 41.10% (95%CI 37.70 to 44.32%) in PG-SGA SF > 5 subgroup and resulting in the rate of 323.98 events per 1000 patient-years. The relationship between the results of nutritional assessment and clinicopathological features of the studied patients is summarized in supplementary Fig. 3. Worsening malnutrition status was associated with a higher incidence of all-cause mortality regardless of the nutritional assessment tool (Fig. 1 and Supplementary Fig. 4).

All nutritional assessment tools were correlated with each other (PG-SGA SF vs. PG-SGA: r = 0.98; PG-SGA SF vs. GLIM: r = 0.48; PG-SGA vs. GLIM: r = 0.5). Malnutrition was associated with higher age, lower BMI, more advanced TNM stage, lower A/G, and higher NLR (all *p* < 0.05, Table 1). For the elderly patients diagnosed with malnutrition, the malnutrition status was associated with a high EORTC QLQ-C30 score (supplementary Table 4), the most frequent nutrition impact symptoms were loss of appetite (44.80%), pain (18.10%), nausea (17.60%) (Supplementary Table 5).

**Table 1:** Baseline Characteristics of the Study Population Classified by nutritional status

	Overall	Non-malnutrition	Malnutrition	*p* value
	*n* = 2724	*n* = 1866	*n* = 858	
**Demographic and anthropometric data**				
Gender,male	1778(65.3%)	1197(64.1%)	581(67.7%)	0.076
Age,years	71.0(5.36)	70.6(5.15)	71.9(5.68)	< 0.001
Height,cm	163(8.05)	163(8.14)	163(7.86)	0.135
Weight,kg	59.7(10.8)	61.2(10.6)	56.3(10.6)	< 0.001
BMI,kg/m^2^	22.5(3.53)	23.1(3.41)	21.1(3.40)	< 0.001
< 18.5	349 (12.8%)	155 (8.31%)	194 (22.6%)	
18.5 ~ 24	1478 (54.3%)	979 (52.5%)	499 (58.2%)	< 0.001
> 24	897 (32.9%)	732 (39.2%)	165 (19.2%)	
CC,cm	32.5(4.12)	33.1(4.01)	31.3(4.12)	< 0.001
HGS,kg	22.5(9.24)	23.4(8.91)	20.5(9.63)	< 0.001
**Risk factors and prior disease**				
Chronic Disease,yes	126(4.63%)	80(4.29%)	46(5.36%)	0.254
Smoking				
Never	1421(52.2%)	976(52.3%)	445(51.9%)	0.18
Now	900(33.0%)	629(33.7%)	271(31.6%)	
Used	403(14.8%)	261(14.0%)	142(16.6%)	
Alcohol,yes	547(20.1%)	366(19.6%)	181(21.1%)	0.398
Tumors				
Lung cancer	791(29.0%)	593(31.8%)	198(23.1%)	< 0.001
Digestive system cancer	1245(45.7%)	738(39.5%)	507(59.1%)	
Others^a^	688(25.3%)	535(28.7%)	153(17.8%)	
Tumor stage				
I	298(10.9%)	243(13.0%)	55(6.41%)	< 0.001
II	642(23.6%)	467(25.0%)	175(20.4%)	
III	667(24.5%)	453(24.3%)	214(24.9%)	
IV	1117(41.0%)	703(37.7%)	414(48.3%)	
Surgery				
Never	978(35.9%)	642(34.4%)	336(39.2%)	0.015
Used	1025(37.6%)	734(39.3%)	291(33.9%)	
Prepare	721(26.5%)	490(26.3%)	231(26.9%)	
Radiotherapy,yes	417(15.3%)	279(15.0%)	138(16.1%)	0.481
Chemotherapy,yes	1455(53.4%)	1044(55.9%)	411(47.9%)	< 0.001
Immunotherapy,yes	133(4.88%)	103(5.52%)	30(3.50%)	0.029
**Laboratory data**				
Creatinine,μmol/L	74.5(33.9)	74.3(33.6)	74.9(34.6)	0.647
A/G	1.34(0.33)	1.38(0.33)	1.25(0.31)	< 0.001
NLR **≥ 3**	1173(43.1%)	694(37.2%)	479(55.8%)	< 0.001
**Assessment**				
KPS,> 70	456(16.7%)	163(8.74%)	293(34.1%)	< 0.001

Values are mean(standard deviation) or n (%)

*BMI* Body Mass Index, *TSF* Triceps Skin Fold, *CC* calf circumference, *HGS* hand grip strength, *A/G* Albumin globulin ratio, *NLR* Neutrophil To Lymphocyte Ratio, *KPS* Karnofsky Performance Status. Chronic Disease: with with one or more chronic conditions (including Hepatitis, or cirrhosis, or renal dialysis patients, or chronic obstructive pulmonary disease, or pulmonary tuberculosis). Tumors, Others^a^: Including breast cancer, cervical cancer, ovarian cancer, endometrial cancer, bladder cancer, prostatic cancer, and nasopharynx cancer

### Comparative performance and validation of the PG-SGA SF

**Fig. 2:**
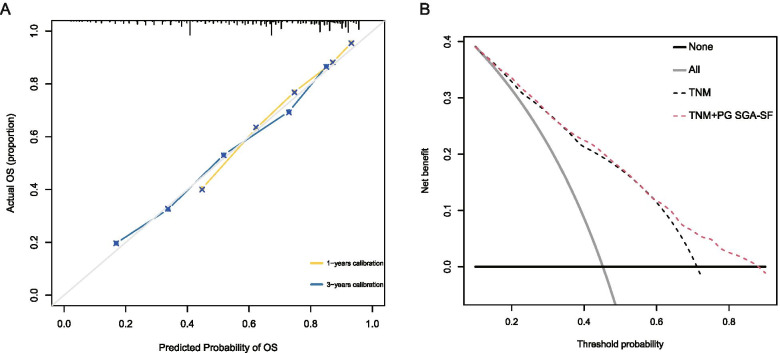
calibration plot and decision curve analysis for PG-SGA SF. **A** Calibration curves of the TNM stage combined with PG-SGA SF model. **B** Decision curve analysis on the TNM stage (black line), and TNM stage combined with PG-SGA SF (red line). Gray line denotes the assumption that all patients have outcome event (death) during follow-up. Thick black line represents the assumption that no patients have outcome event (death) during follow-up

The time-AUCs of the PG-SGA SF for predicting the overall survival rates exhibited similar survival predictive ability with that of the PG-SGA but were significantly higher than that of the GLIM system (Supplementary Fig. 5A). For mortality risk prediction, PG-SGA SF provided a significant incremental prognostic value on the TNM classification system, as was seen using Harrell’s concordance index and ROC curves (Table 2 and Supplementary Fig. 5B). The PG-SGA SF gave rise to a new c-index of 0.739(95% CI: 0.724–0.753) compared with the previous value of 0.700(95% CI: 0.686–0.713) for the TNM classification system. The calibration curves revealed high agreement between the predicted probability of OS and actual observed survival in 1- and 3- years (Fig. 2A). Besides, the DCA curves showed that the PG-SGA SF combined with the TNM classification system had better benefits than the TNM classification system (Fig. 2B).

**Table 2:** Model Performance After the Addition of PG-SGA SF to the TNM classification system for Predicting All-Cause Mortality

	c-statistic	cNRI	IDI
TNM	0.700(0.686, 0.713)	vs.	vs.
TNM & PG-SGA-SF	0.739(0.724, 0.753)	0.125	0.043
*P* value	< 0.001	< 0.001	< 0.001

*cNRI* continuous net reclassification improvement, *IDI* integrated discrimination improvement, *PG-SGA SF* Scored Patient-Generated Subjective Global Assessment Short form

When stratified by tumor type, the PG-SGA SF was consistently associated with worse OS in elderly patients with respiratory system tumors, digestive system tumors, and other tumors (Supplementary Fig. 6). Furthermore, Multivariate analysis indicated that the PG-SGA SF maintained an independent prognostic factor of OS for elderly patients with cancer in different tumor types (supplementary Table 6). When stratified by the TNM stage, the PG-SGA SF could allow for identifying a significant distinction in the Kaplan-Meier curves for survival outcomes (Supplementary Fig. 7).

### Sensitive analysis

As a sensitivity analysis, we fit models adjusting for excluding patients dying within 1 year or excluding patients with chronic disease. Consistently, the results were similar to when those patients were included (adjusted HR 1.32, 95% CI: 1.13–1.55 for excluding patients dying within 1 year; adjusted HR 1.91, 95% CI: 1.02–3.58 for excluding patients with chronic disease). Additionally, a small proportion of patients received immunotherapy in this study data set (*n* = 133, 4.88%). A sensitivity analysis on the effect of immunotherapy in the prognosis of the PG-SGA SF performed that patients with malnutrition had a significantly worse OS than patients without malnutrition (adjusted HR 2.56, 95% CI: 1.33–4.95) (Table 3 and Supplementary Fig. 8).

**Table 3:** Hazard risk for all cause mortality in elder patients by excluding patients dying within 1 years or patients with chronic disease or patients treated with immunotherapy

	HR (95%CI)		HR (95%CI)^a^	*p* value
Excluding patients dying within 1 years				
PG-SGA SF(as continuous)	1.06(1.05,1.08)	< 0.001	1.02 (1.01,1.04)	0.008
PG-SGA SF				
≤ 5	ref		ref	
> 5	1.61(1.39,1.87)	< 0.001	1.32 (1.13,1.55)	0.001
Excluding patients with chronic disease				
PG-SGA-SF(as continuous)	1.12(1.07,1.18)	< 0.001	1.09 (1.02,1.17)	0.008
PG-SGA-SF				
≤ 5	ref		ref	
> 5	2.67(1.62,4.39)	< 0.001	1.91 (1.02,3.58)	0.044
Patients treated with immunotherapy.				
PG-SGA-SF(as continuous)	1.13(1.07,1.2)	< 0.001	1.10(1.02,1.17)	0.008
PG-SGA-SF				
≤ 5	ref			
> 5	2.63(1.56,4.44)	< 0.001	2.56(1.33,4.95)	0.005

Abbreviations: *HR* hazard ratio, *PG-SGA-SF* Scored Patient-Generated Subjective Global Assessment Short form

^a^ Adjusted by: gender, age, smoking, alcohol, tumors type, TNM stage, surgery, radiotherapy, chemotherapy, KPS, A/B, NLR, HGS

## Discussion

Due to the high heterogeneity of solid tumors, it is difficult to accurately predict a patient’s survival, even using the TNM staging system [3]. There is substantial evidence that nutritional status influences the cancer patient’s survival. This study demonstrated that the optimal cut-off scores of PG-SGA SF were five for the elderly patients with cancer,consistent with the previous research [21]. Malnutrition is common in elderly patients with cancer.It is associated with poor prognostic regardless of the existing malnutrition assessment tools used, TNM stage, tumor types, treatment method, and other risk factors. Our study also identified the capability of using PG-SGA SF in determining OS in elderly patients with cancer.

Nutritional assessment is one of the essential features of the comprehensive geriatric oncology assessment to predict mortality [24]. In the current study, the researchers found that the PG-SGA SF is a suitable nutrition assessment tool for elderly patients with cancer and is an excellent alternative to the PG-SGA and GLIM. Since the PG-SGA SF was designed to be completed by the patient with most questions that are easy to understand, it was relatively easy to complete. It could save time for both healthcare and patients and could improve patients’ autonomy [21]. The PG-SGA SF included four significant domains (weight history, food intake, nutrition symptoms, and physical function). Compared to GLIM, the PG-SGA SF identified more patients at a status of malnutrition (31.5% vs. 27.6%). This could be explained as the nutrition impact symptoms were included in the PG-SGA SF.

Unintentional weight loss is an important criterion when assessing nutritional status in cancer patients. It is often the first visible sign of the disease among patients with cancer, with 40% of the patients reporting that they had lost more than 10% of their usual body weight when the first diagnosed [25]. Reduce intake and nutrition impact symptoms were common in the current study, with most of the patients reporting at least one symptom in the past few months. The intervention of nutrition impact symptoms, especially early in the patients with no significant weight loss, may facilitate malnutrition prevention and the improved quality of life. Furthermore, geriatric oncology interventions mostly aimed to address problems in thequality of life, nutritional status, and OS [26]. This study and previous research also found that the quality of life was poorer among patients with malnutrition compared to patients without malnutrition [27]. Additionally, it is noteworthy that worse functional capacity is frequently observed in elderly patients with cancer due to tumor burden, hypercatabolism, reduced food intake, cancer treatment, and decreased physical activity.

Recently studies demonstrated that malnutrition could be associated with reduced treatment effectiveness, functional status, quality of life, and survive [21]. What’s more, in our recent study, malnutrition is associated with a worse response of immunotherapy in elderly patients with cancer [28]. A possible explanation is that the lymphocyte is a sub-clinical biomarker of nutrition, as the total lymphocyte count is decreased in cases of malnutrition [29]. As mentioned earlier, there is evidence that elderly patients are inherently at risk of malnutrition. A crucial and challenging issue in geriatric oncology is to consider whether malnutrition is the consequence of cancer or previous comorbidities (or chronic conditions). Some chronic conditions may influence the status of nutritional, but not survival [30]. We found that malnutrition remains an independent prognostic factor by independently analyzing elderly patients without chronic conditions.

The limitation of this study is that weight change is not an objective indicator of disease status in the presence of ascites, edema, or the growth of the tumor itself (including its metastases). Therefore, an evaluation of body weight instead of body composition can be misleading [31]. Additionally, the nutritional assessment was conducted only at the start of admission; we did not investigate the changes in nutritional status over time. Finally, additional confounding factors such as early deaths relative to specific treatment toxicity and deaths from no-tumor causes were not considered. But anyhow, this study also has several strengths. There is little known about the impact of nutritional status of elderly patients with cancer in Asia on patient-related outcomes, such as quality of life and OS. In addition, we confirmed that the PG-SGA SF qualifies as an independent, convenient, and universally available assessment tool to predict prognosis in elderly patients with cancer. When combined with the TNM classification system, it may represent a more accurate prognostic.

## Conclusions and implications

In conclusion, our study showed that the PG-SGA SF is strongly correlated with outcomes in elderly patients with cancer. Notably, these characteristics of PG-SGA SF are commonly assessed in daily clinical practice in hospitalized patients, which is a practical advantage. Adequate assessment of nutritional status could help improve the prognosis of elderly patients with cancer and select those patients who may benefit from nutritional support. Oncologists should consider this factor as a part of comprehensive geriatric assessment before recommending different treatments for elderly patients with cancer. Future research is still necessary to the PG-SGA SF effectiveness in elderly patients with cancer.

## Supplementary Information

**Additional file 1: Supplementary Figure 1.** A flow chart of the patients inclusion. **Supplementary Figure 2.** Estimation of the cut-off value for the PG-SGA SF. **Supplementary Figure 3.** Correlation analysis of clinical parameters. The blue and red edges represent negative and positive correlations, respectively, and the stronger is in correlation, the darker in color. **Supplementary Figure 4.** Kaplan-Meier curves for all-cause mortality by the PG-SGA and GLIM in elderly patients with cancer. **Supplementary Figure 5.** (A)Time-dependent area under the curve (AUC) by the three nutritional assessment tools. (B) Time-dependent ROC curves for survival prediction in 12 months by TNM stage model and TNM stage model combined with PG-SGA SF. **Supplementary Figure 6.** Kaplan-Meier curves for all-cause mortality by the PG-SGA SF in different tumor types. **Supplementary Figure 7.** Kaplan-Meier curves for all-cause mortality by the PG-SGA SF in each TNM stages. **Supplementary Figure 8.** Kaplan-Meier curves for all-cause mortality by the PG-SGA SF in elderly patients with cancer treatment with immunotherapy.

## Data Availability

All data needed to evaluate the conclusions in the paper are present in the paper and/or the Supplementary Materials. Data described in the manuscript, code book, and analytic code will be made available upon request pending application and approval.
