# Supplementary material for: PG-SGA SF in nutrition assessment and survival prediction for elderly patients with cancer
Source: BMC Geriatr. 2021 Dec 10;21:687. doi: 10.1186/s12877-021-02662-4 (PMC8665602; doi:10.1186/s12877-021-02662-4)

Supplementary table 1 Baseline Characteristics of the Study Population classified by gender.

|  | Overall | Male | Female | p value |
| --- | --- | --- | --- | --- |
|  | n=2724 | n=1778 | n=946 |  |
| **Demographic and anthropometric data** | | |  |  |
| Age ,years | 71.0(5.36) | 71.1(5.34) | 70.7(5.36) | 0.03 |
| Height ,cm | 163(8.05) | 167(6.23) | 156(5.96) | <0.001 |
| Weight, kg | 59.7(10.8) | 62.0(10.4) | 55.3(10.2) | <0.001 |
| BMI ,kg/㎡ | 22.5(3.53) | 22.3(3.35) | 22.8(3.82) | 0.001 |
| CC ,cm | 32.5(4.12) | 32.8(4.18) | 32.0(3.97) | <0.001 |
| HGS ,kg | 22.5(9.24) | 25.5(9.02) | 16.8(6.56) | <0.001 |
| **Risk factors and prior disease** | | |  |  |
| Chronic Disease ,yes | 126(4.63%) | 88(4.95%) | 38(4.02%) | 0.314 |
| Smoking |  |  |  | <0.001 |
| Never | 1421(52.2%) | 579(32.6%) | 842(89.0%) |  |
| Now | 900(33.0%) | 811(45.6%) | 89(9.41%) |  |
| Used | 403(14.8%) | 388(21.8%) | 15(1.59%) |  |
| Alcohol ,yes | 547(20.1%) | 534(30.0%) | 13(1.37%) | <0.001 |
| Tumors |  |  |  | <0.001 |
| Lung cancer | 791(29.0%) | 566(31.8%) | 225(23.8%) |  |
| Digestive system cancer | 1245(45.7%) | 886(49.8%) | 359(37.9%) |  |
| Others | 688(25.3%) | 326(18.3%) | 362(38.3%) |  |
| Tumor stage |  |  |  | <0.001 |
| I | 298(10.9%) | 153(8.61%) | 145(15.3%) |  |
| II | 642(23.6%) | 398(22.4%) | 244(25.8%) |  |
| III | 667(24.5%) | 455(25.6%) | 212(22.4%) |  |
| IV | 1117(41.0%) | 772(43.4%) | 345(36.5%) |  |
| Surgery |  |  |  | <0.001 |
| Never | 978(35.9%) | 682(38.4%) | 296(31.3%) |  |
| Used | 1025(37.6%) | 600(33.7%) | 425(44.9%) |  |
| Prepare | 721(26.5%) | 496(27.9%) | 225(23.8%) |  |
| Radiotherapy ,yes | 417(15.3%) | 281(15.8%) | 136(14.4%) | 0.353 |
| Chemotherapy ,yes | 1455(53.4%) | 946(53.2%) | 509(53.8%) | 0.796 |
| Immunotherapy ,yes | 133(4.88%) | 77(4.33%) | 56(5.92%) | 0.082 |
| **Laboratory data** |  |  |  |  |
| Creatinine ,μmol/L | 70.0(59.0;84.0) | 75.7(65.1;88.0) | 60.2(52.6;70.0) | <0.001 |
| A/G | 1.34(0.33) | 1.32(0.30) | 1.37(0.37) | 0.001 |
| NLR | 1173(43.1%) | 819(46.1%) | 354(37.4%) | <0.001 |
| **Assessment** |  |  |  |  |
| KPS ,>70 | 456(16.7%) | 295(16.6%) | 161(17.0%) | 0.818 |
| PGSGA-SF , >5 | 858(31.5%) | 581(32.7%) | 277(29.3%) | 0.076 |
| PGSGA , >3 | 1759(64.6%) | 1139(64.1%) | 620(65.5%) | 0.468 |
| GLIM, positive | 752(27.6%) | 501(28.2%) | 251(26.5%) | 0.385 |
| EORTC-QLQ-C30 | 39.5 (36.6;42.2) | 39.1 (36.4;42.1) | 39.9 (36.7;42.4) | 0.013 |

BMI: Body Mass Index, TSF: Triceps Skin Fold, MAMC: mid-arm muscle circumference, CC: calf circumference, HGS: hand grip strength, A/G: Albumin globulin ratio, NLR: Neutrophil To Lymphocyte Ratio, KPS: Karnofsky Performance Status, PGSGA-SF: Scored Patient-Generated Subjective Global Assessment Short form, PG-SGA: Scored Patient-Generated Subjective Global Assessment, GLIM: Global Leadership Initiative on Malnutrition

Supplementary table 2 Clinico-pathological characteristics in elder patients: univariate and multivariate survival analysis.

|  |  | Univariate analysis | | Multivariate analysis | |
| --- | --- | --- | --- | --- | --- |
|  | Event(%) | HR (95%CI) | p value | HR (95%CI) | p value |
| Gender |  |  |  |  |  |
| Male | 833(70.80%) | Ref. |  | Ref. |  |
| Female | 343(29.20%) | 0.67(0.59,0.77) | <0.001 | 0.53(0.20,1.39) | 0.199 |
| Age ,years(per 10 years ) |  | 1.33(1.20,1.47) | <0.001 | 1.00(0.89,1.13) | 0.966 |
| Height ,cm |  | 1.01(1.00,1.02) | 0.028 |  |  |
| Weight ,kg |  | 0.98(0.97,0.98) | <0.001 |  |  |
| BMI ,kg/㎡ |  | 0.92(0.90,0.93) | <0.001 | 1.02(0.92,1.13) | 0.699 |
| CC ,cm |  | 0.94(0.93,0.95) | <0.001 | 1.00(0.98,1.02) | 0.773 |
| HGS ,kg. |  | 0.98(0.97,0.99) | <0.001 | 0.98(0.98,0.99) | <0.001 |
| Chronic Disease ,yes |  |  |  |  |  |
| Absent | 1113(94.60%) | Ref. |  |  |  |
| Present | 63(5.36%) | 1.17(0.90,1.50) | 0.238 |  |  |
| Smoking |  |  |  |  |  |
| Never | 555(47.20%) | Ref. |  |  |  |
| Now | 433(36.80%) | 1.28(1.13,1.46) | <0.001 | 1.03(0.88,1.19) | 0.740 |
| Used | 188(16.00%) | 1.36(1.15,1.61) | <0.001 | 0.92(0.76,1.12) | 0.415 |
| Alcohol |  |  |  |  |  |
| Absent | 914(77.70%) | Ref. |  |  |  |
| Present | 262(22.30%) | 1.23(1.07,1.41) | 0.004 | 1.13(0.97,1.31) | 0.129 |
| Tumors |  |  | <0.001 |  |  |
| Lung cancer | 438(37.20%) | Ref. |  |  |  |
| Digestive system cancer | 526(44.70%) | 0.66(0.58,0.75) | <0.001 | 0.84(0.72,0.98) | 0.027 |
| Others | 212(18.00%) | 0.48(0.41,0.57) | <0.001 | 0.61(0.51,0.73) | <0.001 |
| Tumor stage |  |  |  |  |  |
| I | 37(3.15%) | Ref. |  |  |  |
| II | 147(12.50%) | 2.01(1.40,2.88) | <0.001 | 1.83(1.27,2.63) | 0.001 |
| III | 256(21.80%) | 3.80(2.69,5.37) | <0.001 | 3.11(2.19,4.4) | <0.001 |
| IV | 736(62.60%) | 9.85(7.07,13.7) | <0.001 | 6.54(4.63,9.23) | <0.001 |
| Surgery |  |  |  |  |  |
| Never | 608(51.70%) | Ref. |  | Ref. |  |
| Used | 376(32.00%) | 0.39(0.34,0.45) | <0.001 | 0.70(0.6,0.82) | <0.001 |
| Prepare | 192(16.30%) | 0.31(0.26,0.36) | <0.001 | 0.70(0.57,0.87) | 0.001 |
| Radiotherapy |  |  |  |  |  |
| No | 976(83.00%) | Ref. |  | Ref. |  |
| Yes | 200(17.00%) | 1.30(1.11,1.51) | 0.001 | 1(0.85,1.17) | 0.979 |
| Chemotherapy |  |  |  |  |  |
| No | 462(39.30%) | Ref. |  | Ref. |  |
| Yes | 714(60.70%) | 1.50(1.34,1.69) | <0.001 | 1.06(0.92,1.23) | 0.394 |
| Immunotherapy |  |  |  |  |  |
| No | 1110(94.40%) | Ref. | Ref. |  |  |
| Yes | 66(5.61%) | 1.10(0.85,1.41) | 0.470 |  |  |
| Creatinine |  | 1.01(1.00,1.03) | 0.113 |  |  |
| A/G | 1.26(0.36) | 0.26(0.22,0.32) | <0.001 | 0.57(0.47,0.71) | <0.001 |
| NLR |  |  |  |  |  |
| <3 | 537(45.7%) | Ref. |  | Ref. |  |
| >3 | 639(54.3%) | 1.87(1.67,2.10) | <0.001 | 1.40(1.24,1.58) | <0.001 |
| KPS |  |  |  |  |  |
| ≤70 | 878(74.7%) | Ref. |  | Ref. |  |
| >70 | 298(25.3%) | 2.11(1.85,2.41) | <0.001 | 1.39(1.19,1.62) | <0.001 |
| PG-SGA SF |  |  |  |  |  |
| ≤5 | 670(57.00%) | Ref. |  | Ref. |  |
| >5 | 506(43.00%) | 2.10(1.87,2.35) | <0.001 | 1.47(1.29,1.68) | <0.001 |
| EORTC-QLQ-C30 |  | 1.00(0.99,1.01) | 0.832 |  |  |

Abbreviations: HR, hazard ratio; BMI: Body Mass Index, TSF: Triceps Skin Fold, CC: calf circumference, HGS: hand grip strength, A/G: Albumin globulin ratio, NLR: Neutrophil To Lymphocyte Ratio, KPS: Karnofsky Performance Status, PG-SGA-SF: Scored Patient-Generated Subjective Global Assessment Short form. Chronic Disease: with with one or more chronic conditions (including Hepatitis, or cirrhosis, or renal dialysis patients, or chronic obstructive pulmonary disease, or pulmonary tuberculosis).

Supplementary table 3 Cox Proportional Hazards Analyses of PG-SGA and GLIM to Predict All-Cause Mortality.

|  | Event(%) | HR (95%CI) | p value | HR (95%CI)^a^ | p value |
| --- | --- | --- | --- | --- | --- |
| **PG-SGA** |  |  |  |  |  |
| ≤3 | 302(25.7%) | Ref. |  | Ref. |  |
| >3 | 874(74.3%) | 1.79(1.57;2.03) | <0.001 | 1.31(1.13,1.51) | <0.001 |
| **GLIM** |  |  |  |  |  |
| non-malnutrition | 762(64.8%) | Ref. |  | Ref. |  |
| malnutrition | 414(35.2%) | 1.83(1.62;2.07) | <0.001 | 1.24(1.07,1.44) | 0.005 |

Abbreviations: HR, hazard ratio; PG-SGA: Scored Patient-Generated Subjective Global Assessment, GLIM: Global Leadership Initiative on Malnutrition.

^a^ Adjusted by: gender, age, smoking, alcohol, tumors type, TNM stage, surgery, radiotherapy, chemotherapy, KPS, A/B, NLR, HGS.

Supplementary table 4 Quality of life stratified by nutritional status.

|  | overall | Non-malnutrition | malnutrition | P value |
| --- | --- | --- | --- | --- |
|  | n=2724 | n=1866 | n=858 |  |
| Physical function | 86.7(73.3;100) | 93.3(80.0;100) | 80.0(53.3;93.3) | <0.001 |
| Role function | 83.3(66.7;100) | 100(66.7;100) | 66.7(33.3;100) | <0.001 |
| Emotional function | 100(83.3;100) | 100(83.3;100) | 91.7(66.7;100) | <0.001 |
| Congnitive function | 100(83.3;100) | 100(83.3;100) | 83.3(66.7;100) | <0.001 |
| Social function | 66.7(66.7;100) | 83.3(66.7;100) | 66.7(50.0;100) | <0.001 |
| Global QOL | 66.7(50.0;75.0) | 66.7(50.0;83.3) | 50.0(33.3;66.7) | <0.001 |
| Fatigue | 11.1(0.00;33.3) | 11.1(0.00;22.2) | 33.3(11.1;44.4) | <0.001 |
| Nausea and vomiting | 0.00(0.00;0.00) | 0.00(0.00;0.00) | 0.00(0.00;16.7) | <0.001 |
| Pain | 0.00(0.00;16.7) | 0.00(0.00;16.7) | 16.7(0.00;33.3) | <0.001 |
| Dyspnea | 0.00(0.00;33.3) | 0.00(0.00;0.00) | 0.00(0.00;33.3) | <0.001 |
| Sleep disturbance | 0.00(0.00;33.3) | 0.00(0.00;33.3) | 33.3(0.00;33.3) | <0.001 |
| AP | 0.00(0.00;33.3) | 0.00(0.00;0.00) | 33.3(0.00;33.3) | <0.001 |
| Appetite loss | 0.00(0.00;0.00) | 0.00(0.00;0.00) | 0.00(0.00;33.3) | <0.001 |
| Diarrhea | 0.00(0.00;0.00) | 0.00(0.00;0.00) | 0.00(0.00;0.00) | <0.001 |
| Financial difficulties | 33.3(0.00;33.3) | 33.3(0.00;33.3) | 33.3(0.00;66.7) | <0.001 |
| EORTC-QLQ-C30 | 39.5(36.6;42.2) | 39.1(36.7;41.4) | 40.3(36.3;44.0) | <0.001 |

Data are represented as median (interquartile range).

Supplementary table 5 The baseline characteristics of the nutrition impact symptoms.

|  | overall | Non-malnutrition | malnutrition | P value |
| --- | --- | --- | --- | --- |
|  | n=2724 | n=1866 | n=858 |  |
| Loss of appetite ,yes | 471(17.3%) | 87(4.66%) | 384(44.8%) | <0.001 |
| Nausea ,yes | 213(7.82%) | 62(3.32%) | 151(17.6%) | <0.001 |
| Emesis ,yes | 140(5.14%) | 12(0.64%) | 128(14.9%) | <0.001 |
| Canker sore ,yes | 23(0.84%) | 13(0.70%) | 10(1.17%) | 0.309 |
| Constipation ,yes | 205(7.53%) | 87(4.66%) | 118(13.8%) | <0.001 |
| Diarrhea ,yes | 94(3.45%) | 25(1.34%) | 69(8.04%) | <0.001 |
| Thirst ,yes | 160(5.87%) | 69(3.70%) | 91(10.6%) | <0.001 |
| No taste ,yes | 139(5.10%) | 38(2.04%) | 101(11.8%) | <0.001 |
| Altered sense of smell ,yes | 52(1.91%) | 11(0.59%) | 41(4.78%) | <0.001 |
| Dysphagia ,yes | 211(7.75%) | 78(4.18%) | 133(15.5%) | <0.001 |
| Flatulence ,yes | 164(6.02%) | 42(2.25%) | 122(14.2%) | <0.001 |
| Pain ,yes | 194(7.12%) | 39(2.09%) | 155(18.1%) | <0.001 |
| Others ,yes | 38(1.40%) | 19(1.02%) | 19(2.21%) | 0.022 |

Supplementary table 6. Hazard risk for cancer special overall survival in elder patients diagnosed with malnutrition by PG-SGA SF (5~).

|  | HR (95%CI)^a^ | p value |
| --- | --- | --- |
| Respiratory system tumor | 1.36 (1.09,1.71) | 0.007 |
| Digestive system tumor | 1.35 (1.11,1.63) | 0.002 |
| Other tumor | 2.42 (1.74,3.37) | <0.001 |

Abbreviations: HR, hazard ratio;

^a^ Adjusted by: gender, age, smoking, alcohol, tumors type, TNM stage, surgery, radiotherapy, chemotherapy, KPS, A/B, NLR, HGS.

Supplementary Figure 1


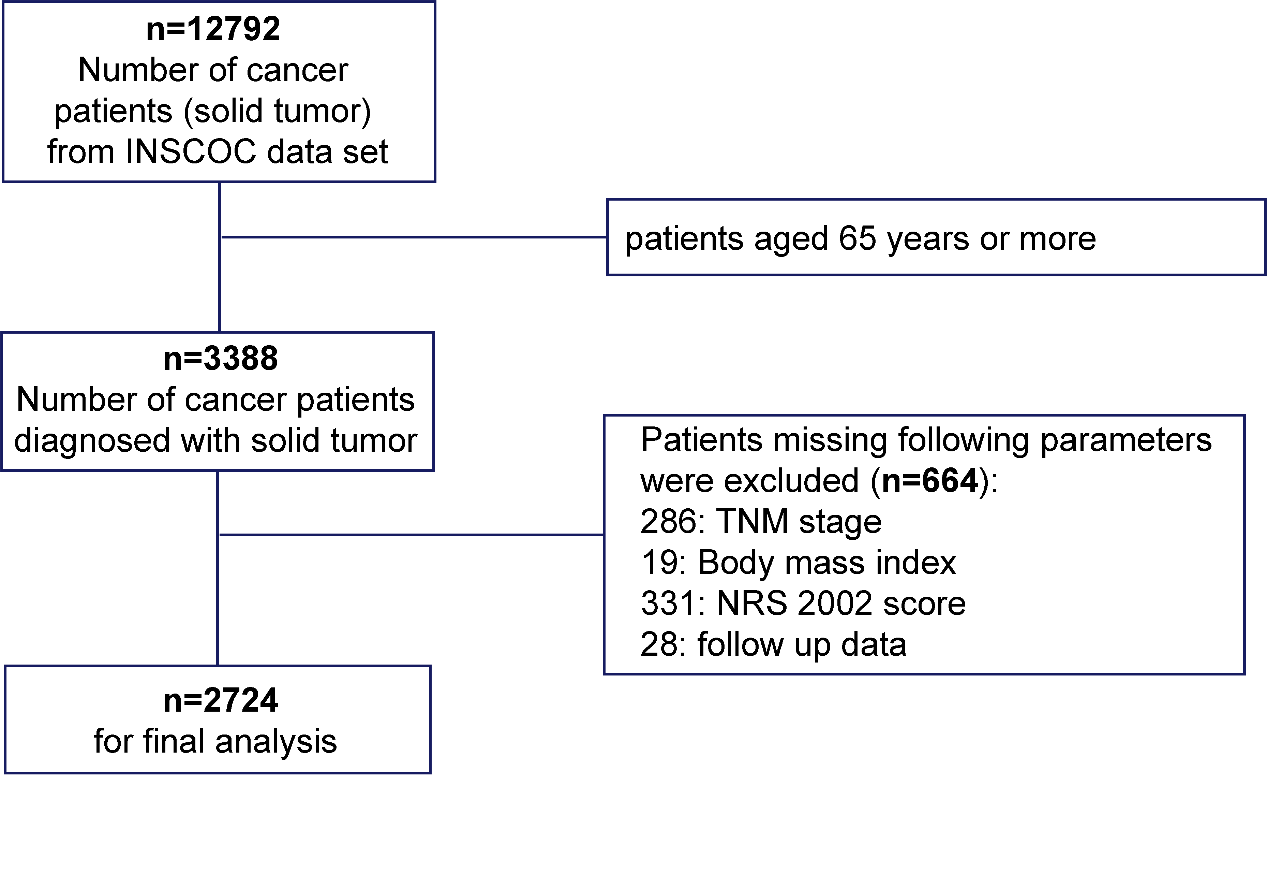


Supplementary Figure 2.


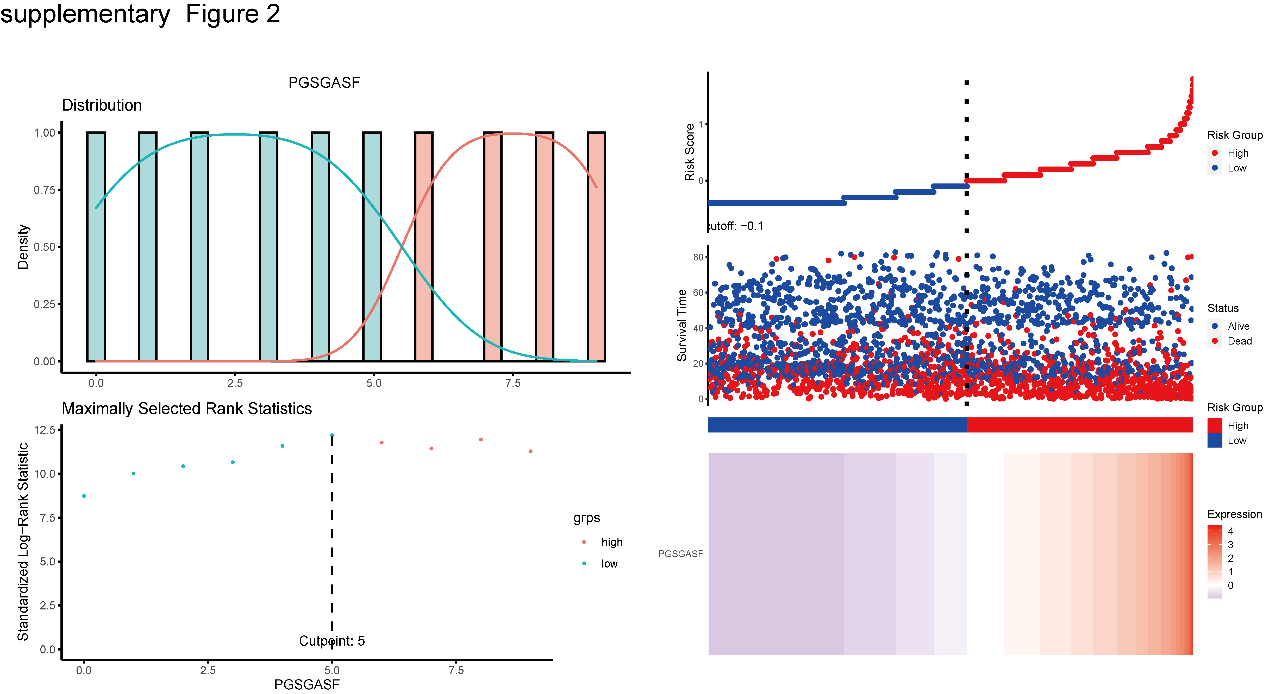


Supplementary Figure 3.


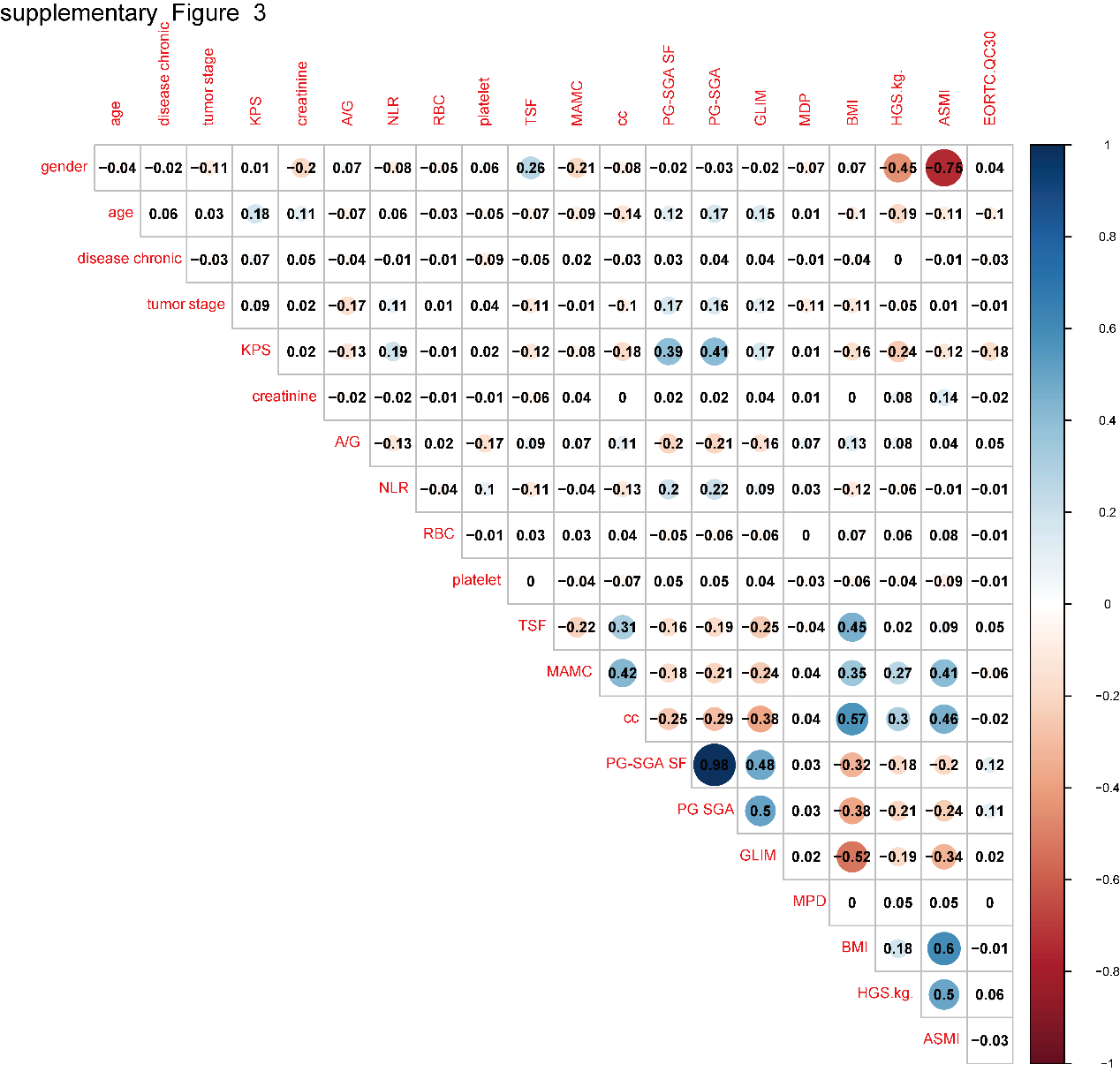


Supplementary Figure 4.


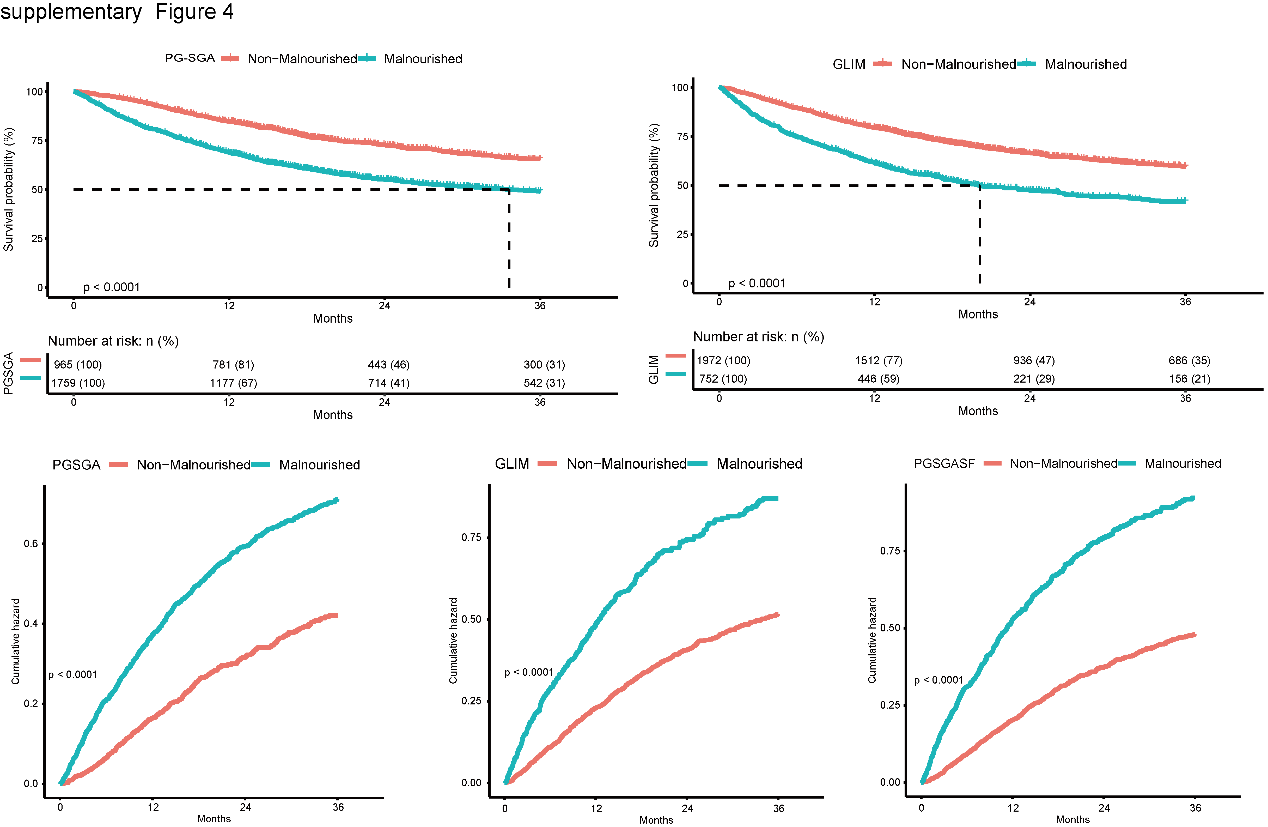


Supplementary Figure 5.


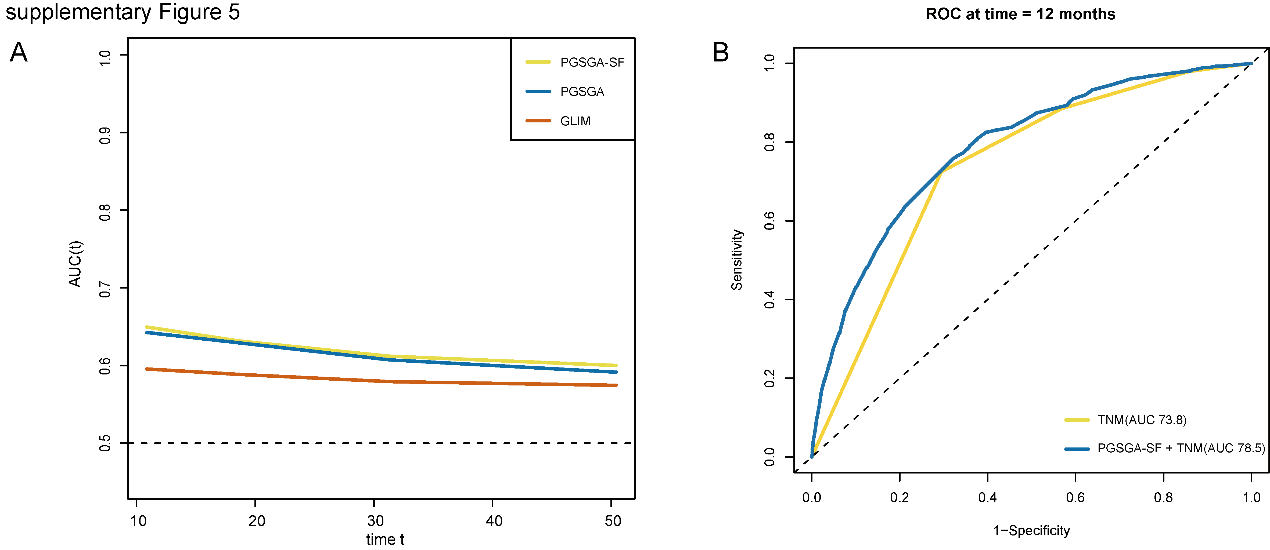


Supplementary Figure 6.


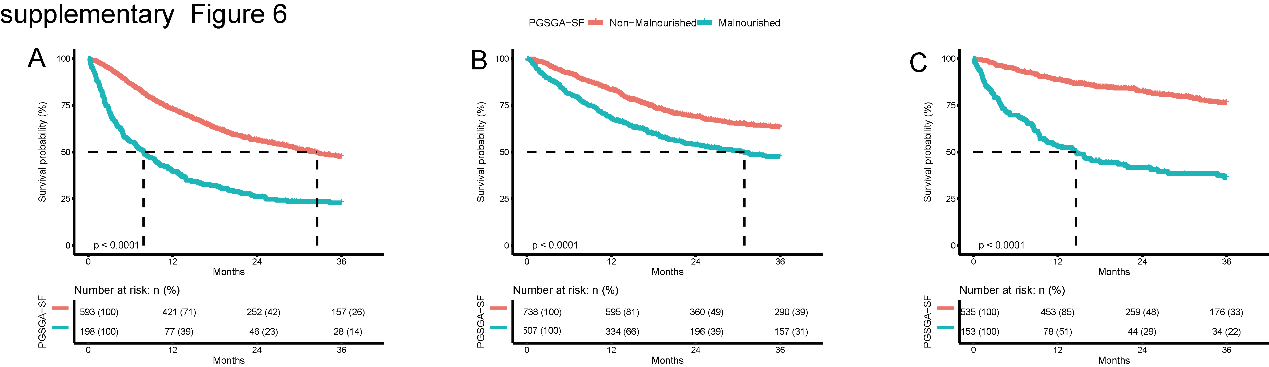


Supplementary Figure 7.


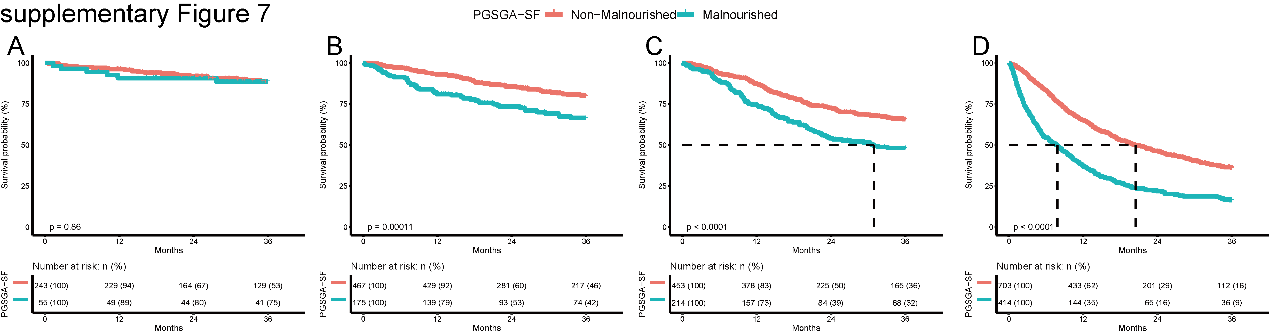


Supplementary Figure 8.


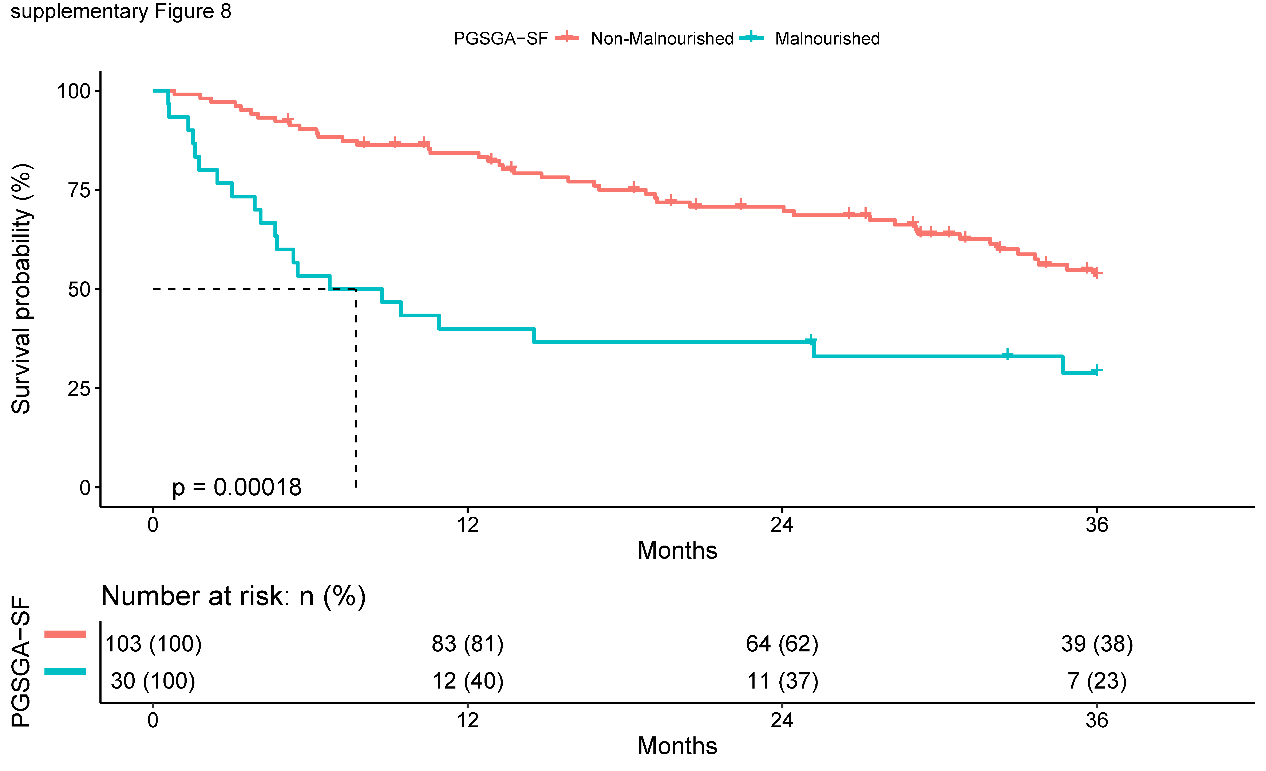

Supplement: Supplementary file 1 — Additional file 1: Supplementary Figure 1. A flow chart of the patients inclusion. Supplementary Figure 2. Estimation of the cut-off value for the PG-SGA SF. Supplementary Figure 3. Correlation analysis of clinical parameters. The blue and red edges represent negative and positive correlations, respectively, and the stronger is in correlation, the darker in color. Supplementary Figure 4. Kaplan-Meier curves for all-cause mortality by the PG-SGA and GLIM in elderly patients with cancer. Supplementary Figure 5. (A)Time-dependent area under the curve (AUC) by the three nutritional assessment tools. (B) Time-dependent ROC curves for survival prediction in 12 months by TNM stage model and TNM stage model combined with PG-SGA SF. Supplementary Figure 6. Kaplan-Meier curves for all-cause mortality by the PG-SGA SF in different tumor types. Supplementary Figure 7. Kaplan-Meier curves for all-cause mortality by the PG-SGA SF in each TNM stages. Supplementary Figure 8. Kaplan-Meier curves for all-cause mortality by the PG-SGA SF in elderly patients with cancer treatment with immunotherapy. [file 12877_2021_2662_MOESM1_ESM.docx]
